# The work of having a chronic condition: development and psychometric evaluation of the distribution of co-care activities (DoCCA) scale

**DOI:** 10.1186/s12913-021-06455-8

**Published:** 2021-05-20

**Authors:** Ulrica von Thiele Schwarz, Marta Roczniewska, Karin Pukk Härenstam, Klas Karlgren, Henna Hasson, Sivan Menczel, Carolina Wannheden

**Affiliations:** 1 School of Health, Care and Social Welfare, Mälardalen University, Box 883, 721 23 Västerås, Sweden; 2 Procome, Medical Management Centre, Department of Learning, Informatics, Management and Ethics (LIME), Karolinska Institutet, 171 77 Stockholm, Sweden; 3 Psychology Department, SWPS University of Social Sciences and Humanities, 81-745 Sopot, Poland; 4 Clinical Management, Medical Management Centre, LIME, Karolinska Institutet, 171 77 Stockholm, Sweden; 5 Paediatric Emergency Department, Astrid Lindgren’s Children’s Hospital, Karolinska University Hospital, 171 76, Stockholm, Sweden; 6 MINT, LIME, Karolinska Institutet, 171 77 Stockholm, Sweden; 7 Department of health and functioning, Faculty of Health and Social Sciences, The Western Norway University of Applied Sciences, 5063 Bergen, Norway; 8 Department of Research, Education, Development and Innovation, Södersjukhuset, 118 83 Stockholm, Sweden; 9 Unit for Implementation and Evaluation, Center for Epidemiology and Community Medicine, Stockholm Region 171 29 Stockholm, Sweden

**Keywords:** Copenhagen Psychosocial Quesionnaire (COPSOQ), Co‐production, Chronic care management, Health and welfare technology, Human‐computer interaction, Patient experience, Patient engagement, Patient participation, Patient preference, Person‐centered care

## Abstract

**Background:**

Chronic care involves multiple activities that can be performed by individuals and healthcare staff as well as by other actors and artifacts, such as eHealth services. Thus, chronic care management can be viewed as a system where the individual interacts with people and eHealth services performing activities to maintain or improve health and functioning, called *co-care*. Yet, the system perspective is not reflected in concepts such as person-centered care and shared decision making. This limits the understanding of individuals’ global experience of chronic care management and subsequently the ability to optimize chronic care. The aim of this study was threefold: (1) to propose a theory-based operationalization of co-care for chronic care management, (2) to develop a scale to measure co-care as a distributed system of activities, and (3) to evaluate the scale’s psychometric properties. With the theory of distributed cognition as a theoretical underpinning, co-care was operationalized along three dimensions: experience of *activities*, *needs support*, and *goal orientation*.

**Methods:**

Informed by the literature on patient experiences and work psychology, a scale denoted Distribution of Co-Care Activities (DoCCA) was developed with the three conceptualized dimensions, the activities dimension consisting of three sub-factors: *demands*, *unnecessary tasks*, and *role clarity*. It was tested with 113 primary care patients with chronic conditions in Sweden at two time points.

**Results:**

A confirmatory factor analysis showed support for a second-order model with the three conceptualized dimensions, with activities further divided into the three sub-factors. Cronbach’s alpha values indicated a good to excellent reliability of the subscales, and correlations across time points with panel data indicated satisfactory test-retest reliability. Convergent, concurrent and predictive validity of the scale were, overall, satisfactory.

**Conclusions:**

The psychometric evaluation supports a model consisting of activities (demands, unnecessary tasks, and role clarity), needs support and goal orientation that can be reliably measured with the DoCCA scale. The scale provides a way to assess chronic care management as a system, considering the perspective of the individuals with the chronic condition and how they perceive the work that must be done, across situations, either by themselves or through healthcare, eHealth, or other means.

**Supplementary Information:**

The online version contains supplementary material available at 10.1186/s12913-021-06455-8.

## Background

Chronic care management takes place 24 h a day, seven days a week and involves many activities that individuals perform themselves to achieve, maintain, or promote health – with or without the support of healthcare services. Such activities include the identification of symptoms, planning treatments, coordinating resources (time and competence), monitoring key health parameters, and assessing progress and treatment effects [1]. In fact, self-care has been recognized as the new principal source of care [2].

The Chronic Care Model [3] acknowledges the necessity to reorganize healthcare services to suit the needs of individuals with chronic conditions. Central to this approach is support for self-care. One enabler of this transformation is the increasing availability and adoption of eHealth services, i.e., health services and information that are delivered or enhanced through the internet and related technologies [4]. eHealth services can be used by individuals to perform activities traditionally performed by healthcare staff [5], which leads to a blurring of boundaries between healthcare and self-care. For example, devices that allow patients to measure, monitor, and share health observations [6] and online patient communities, such as PatientsLikeMe®, where individuals publicly share, compare, and discuss their self-tracked health information, are illustrative examples of how healthcare is reorganized with activities that traditionally took place within patient-provider relationships now being part of a broader system of people and enabling technology.

 We have previously introduced the concept of “co-care” to emphasize the complementary role of healthcare professionals and eHealth in supporting individuals’ resources to achieve favorable health outcomes [7, 8]. This makes co-care a system in which an individual with a chronic condition interacts with people and eHealth to the extent that is necessary to achieve favorable health outcomes. The relationship between people and technology is reciprocal, and the functioning of the system is determined by the system as a whole: individual parts *and* the relationships between them. This makes co-care a sociotechnical system [9]. The co-care system may include healthcare professionals but also others (e.g., family members, peers) who perform activities that contribute to the achievement of the goals and needs of the individual.

The co-care concept has implications regarding how patients’ chronic care management is operationalized and measured. Whereas patient experience has been defined as the sum of all interactions across a continuum of care, it is still restricted to experiences related to healthcare delivery or a certain provider [10]. Existing instruments tend to focus on parts of the co-care system, such as how patients perceive the quality and value of the interaction with healthcare providers [11]. There are also instruments that focus on other, specific aspects of the co-care system, such as how an eHealth service is experienced or a particular care episode (e.g., [12, 13]), despite that many definitions of patient experience emphasize that it cannot be restricted to one encounter [10]. There are also instruments that assess how people experience certain activities, such as the decision-making process [13] and the perceived support to carry out a treatment plan [14]. Patient choice, i.e., the extent to which patients are able to choose between different options, has drawn particular interest [15]. Thus, whereas there are instruments that assess the experience or function of a specific activity or relationship, there is a gap in the literature concerning the experience of all these combined as a *system of activities* that may take place over an extended period of time and situations (i.e., cross-situational) and that can be performed by various actors, including eHealth services [16]. There are also instruments that assess chronic care management according to the chronic care model more generally [17]; however, they reflect the traditional division of tasks between patients and healthcare, illuminating how patients experience the activities that *healthcare* performs. This “inside-out” perspective is shared in much of the patient experience literature [18], leading to a research gap concerning the co-care situation where the division of tasks and roles is not specified.

The assumptions regarding the roles inherent in the patient experience literature are reflected in the use of the term “patient.” The term “patient” defines an individual based on his or her relation to healthcare [19]. This merely captures fragments of an individual’s experience of managing a chronic condition, as defined in co-care. In addition to being a patient, an individual with a chronic condition may also be an eHealth service user, a network member, a self-tracker, etc. There is a need for an instrument that captures all these aspects, reflecting the experience of how the co-care system functions as a whole. Such an approach will expand previous literature investigating specific aspects of chronic care management and the experience in the role as patient. A general assessment of co-care will allow the different parts of the system to be understood in relation to other parts of the system, which can offer valuable insights for the design of healthcare and eHealth services.

### Aim

The aim of this study is threefold: (1) to propose a theory-based operationalization of co-care in chronic care management, (2) to develop a scale to measure co-care as a distributed system of activities, and (3) to evaluate the psychometric properties of the scale.

First, we present a theoretical framework for exploring co-care, and thereafter discuss the operationalization for chronic care. In the methods and results sections, we present the development of the scale and its psychometric evaluation. Finally, the discussion section contrasts the operationalization and the scale to other constructs and instruments and discusses its practical use.

### Operationalization of co‐care

To propose an operationalization of co-care, we draw on the theory of distributed cognition, which is specifically designed to understand the functioning of sociotechnical systems where people interact with each other and technology to achieve a specific goal [20]. The theory describes how cognition is distributed across actors and artifacts (i.e., external representations of information and knowledge) and across space and time. Cognition refers to individuals’ processes, such as attention and memory (e.g., remembering symptoms), representation (e.g., assessment of health status), categorization and causal reasoning (e.g., linking behavior change to health outcomes), decision making, and planning (e.g., coordinating resources and completing tasks) [21]. These types of activities are not performed in isolation in an individual’s mind. For chronic care management, this means that chronic care activities involve interactions between people and eHealth services, where the eHealth services support the collection, processing, storing, sharing, and retrieval of information. Thereby, eHealth services facilitate the distribution of activities over space and time enabling interactions between actors in real-time or asynchronously regardless of their location [4, 7, 21].

**Fig. 1:**
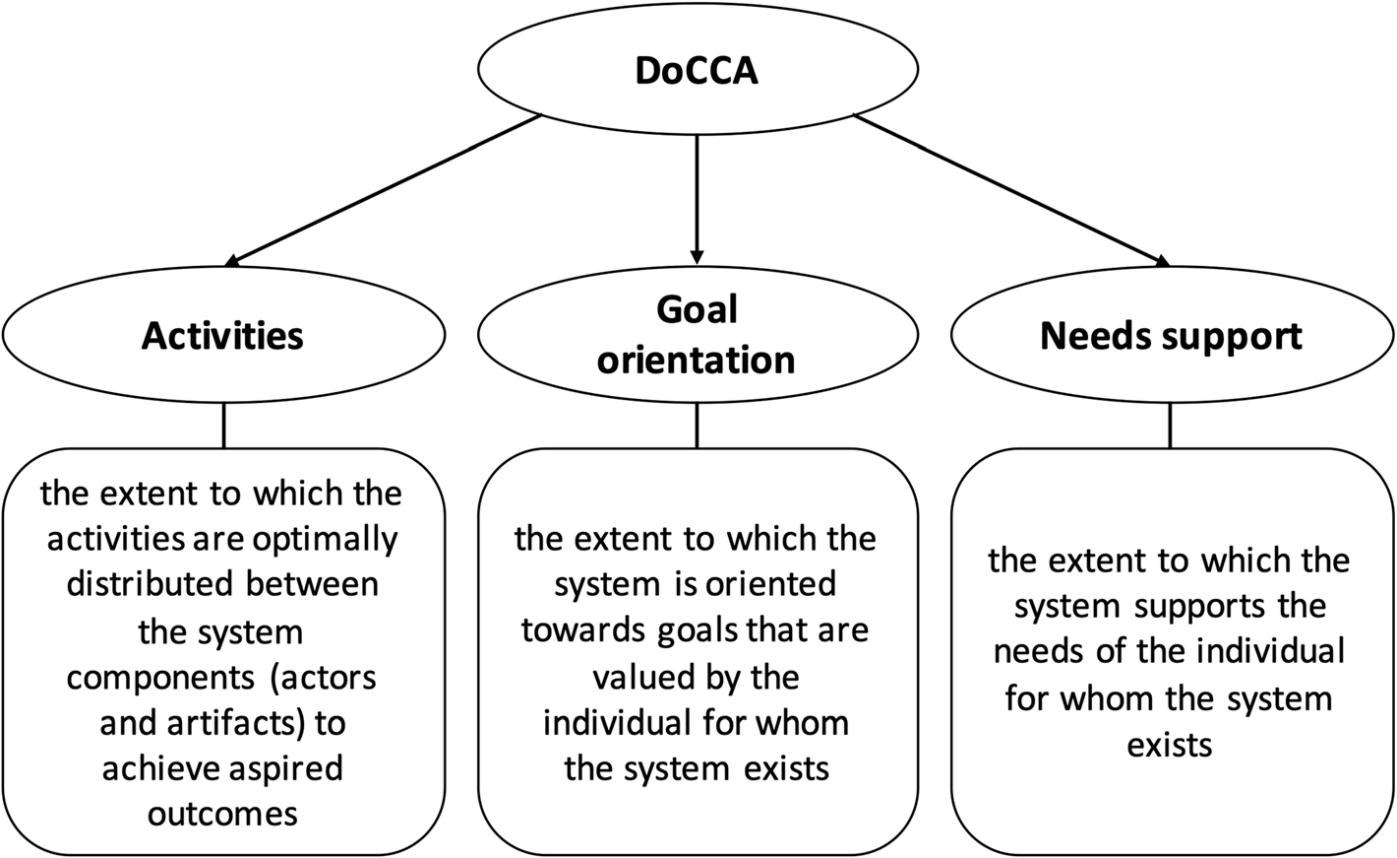
Three dimensions of co-care as a distributed system of activities for management of chronic conditions. DoCCA = Distribution of Co-Care Activities

The operationalization of co-care as a distributed system of activities should reflect the system perspective as defined by the individual for whom the system exists. Acknowledging that individuals make decisions that affect their health 24/7, chronic care activities can be distributed in different ways between the individual, other actors, and artifacts. The (optimal) constellation of actors and artifacts in the system, and in turn the (optimal) distribution of activities, should be determined by the individual for whom the system exists. In other words, the system must be oriented towards the goals that are valued by the individual with the chronic condition and must support his or her needs in striving towards these goals. Consequently, we derive three central dimensions that should be reflected in the operationalization of co-care (Fig. 1).

#### Dimension 1: activities

Co-care emphasizes activities and does not make assumptions about who does what, i.e., roles and responsibilities. Instead, it focuses on the extent to which the division of activities is optimized. The optimal distribution of activities should best support the attainment of a goal given the resources available in the system. The roles of the actors and artifacts are to complement the individual’s resources so that the combined resources maximize goal achievement [8]. This includes finding the distributions that optimize individuals’ empowerment and decision authority while avoiding unreasonable, unrealistic, and sub-optimal demands on individuals [22]. Thus, there is not one ultimate way to optimize the distribution of activities within a system.

Whereas activities have not been central in the patient experience literature, this is a main concern in other research fields, such as computer-supported cooperative work [23], computer-supported collaborative learning [24], and work psychology, where there has been substantial research regarding how individuals perceive the (work) tasks they perform given how tasks are managed (i.e., distributed) in an organization (i.e., a system) [25]. Using work psychology as a lens, chronic care management can be understood as work tasks that need to be done to attain or maintain health goals, thereby drawing attention to the “working conditions” of the co-care system.

A number of factors can explain how people experience their work. They have been summarized in the job demand-resource model, which postulates that the working conditions can be understood as the relationship between perceived demands involved in performing work tasks and the available resources [26]. Demands are the physical, psychological, social, or system aspects of the job (here chronic care management) that require sustained physical and/or psychological effort or skills and that are therefore associated with certain physiological and/or psychological costs. Resources can be personal (e.g., skills and knowledge), interpersonal (e.g., social support), and structural (e.g., infrastructure). Thus, assessing the distribution of activities in chronic care management may entail exploring perceived difficulty of tasks, emotional and cognitive burden, whether the division of tasks is reasonable (or if people are expected to do things they believe others would be better equipped to manage), and whether roles and responsibilities are clear [27, 28]. Therefore, there is no assumption regarding the optimal distribution of activities. Instead, by examining people’s perceived experiences of the distribution, it is acknowledged that individuals may react differently to the same objective distribution, reflecting individual differences in resources, such as life circumstances, health literacy, etc.

#### Dimension 2: goal orientation

The individual with the chronic condition is the only given actor in the system. As such, he or she also determines what the system should accomplish, something that has been described as “Nothing about me without me” [29]. The individual with the chronic condition knows most about the consequences of the chronic condition and treatment in the context of his or her life and must apply this knowledge to guide chronic care management over time [30]. Thus, in a well-functioning, distributed system, the actors co-produce results that are desired by the individual. Examples of this can be found in efforts to design systems that aim to achieve outcomes that matter to patients [31]. This means that an operationalization of co-care must reflect the degree to which the system is optimally designed to achieve the goals that are important to the individual.

Designing the system based on the goals of the individual with the chronic condition entails a shift in perspectives. Rather than asking if the patient is engaged and participates (often without reference to what end), co-care asks if the system as a whole is oriented around the goals of the individual—an *outside-in* perspective [18]. This includes questions about goal awareness, the extent to which goals were co-produced, the alignment of the system with these goals, and whether the chronic care activities are perceived to be oriented towards achieving them. Thus, understanding goal orientation in co-care entails capturing the extent to which *healthcare* is sufficiently aware of the individual’s aspired goals and involved in the fulfillment of these goals.

#### Dimension 3: needs support

A distributed system perspective acknowledges that individuals’ needs can be supported in different ways by both humans and artifacts. Needs support is providing the right help at the right time in the right way [32]. The support can take many different forms: emotional, instrumental, informational, and appraisal [33]. The patient experience literature reflects many of these forms; however, it has focused on how the support from one part of the system (e.g., healthcare) is perceived, such as whether professionals provide accurate and clear information [12, 34] and listen and show respect for the individual’s needs [35]. By widening the perspective to the entire sociotechnical system involved in co-care, needs support can be provided in different ways, such as when significant others coordinate activities [36], by providing health information online [5], by sharing experiences with others, or by self-tracking personal health data [37]. Thus, understanding needs support in co-care entails capturing to what extent people experience that their needs are met regardless of the source.

## Methods

Based on the suggested operationalization of co-care as a distributed system of activities, we set out to develop a scale to measure this and to evaluate the scale’s psychometric properties.

### DoCCA scale development

Initially, several instruments in the patient and user experience literature were scrutinized to identify suitable measures for the operationalization of co-care as a distributed system of activities (e.g., [13, 14, 17, 38–43]). However, as discussed, these did not sufficiently reflect the system perspective of chronic care management and its distributed nature. Thus, we turned to instruments from the work psychology domain, primarily the Copenhagen Psychosocial Questionnaire (COPSOQ) and Bern Illegitimate Tasks Scale (BITS). COPSOQ is a validated instrument to measure psychological work conditions related to work tasks, the organization of work, and interpersonal relations, and has been widely used in both practical settings and in research [44]. BITS measures how individuals perceive the tasks they are expected to perform [45]. It consists of two sub-scales that measure the degree to which tasks are perceived as unnecessary or unreasonable.

#### Item pool generation and selection

An initial item pool was generated from the COPSOQ and BITS instruments through an iterative process. First, we examined all subscales and items for their relevance for the co-care construct (Fig. 1), testing whether they made sense when rephrased to suit the context of chronic care management rather than a work setting (e.g., references to managers, colleagues, and work tasks were rephrased to references relevant to chronic care management viewed from a system perspective). Forty-three candidate items were identified in the first iteration. The number of items were reduced iteratively through discussions among the authors about individual items’ relevance, applicability, acceptability, and appropriateness and the combined coverage of the co-care concept.

This process yielded 14 items, 12 of which were related to distribution of work tasks. They came from three subscales: *Cognitive and emotional demands*, measuring needs to make difficult decisions, remembering or monitoring things, and exposure to emotionally strenuous situations; *Role clarity*, measuring how responsibility for activities is distributed, perceived clarity of responsibilities and expectations, and whether the distribution is perceived as just; *Unnecessary tasks* (from BITS), measuring tasks that do not need to be performed. To reflect the factor structure from the original scales, we thus propose that distribution of activities consists of three sub-factors (demands, role clarity, and unnecessary tasks). We found two items of relevance for needs support, one from *Social support*, assessing whether one receives the support needed, and one from *Predictability*, namely if one receives information needed to perform tasks.

#### Generation of new items and refinement

With only two items reflecting needs support and none of relevance for goal orientation, we returned to the patient satisfaction survey used in Swedish healthcare [46] and adapted two items, one assessing participation in decision making and one for information clarity. Still not finding any items reflecting the perspective shift evident in the conceptualization of the goal orientation of the system, we then created four new items in an iterative process where all authors were engaged.

Feedback from nine individuals (representing individuals with chronic conditions, eHealth-experts, healthcare professionals, and researchers) was elicited at different time points in the development process. We also performed cognitive interviews with three individuals with different chronic conditions (hypertension, chronic pain, diabetes) to assess item comprehension and the relevance of the final version, leading to minor language edits.

#### The DoCCA Scale

The final DoCCA scale consists of 20 items. For all items, a five-point Likert response scale was used, consistent with COPSOQ, with the following anchors: 1 = To a very low degree; 2 = To a low degree; 3 = Partially; 4 = To a high degree; and 5 = To a very high degree. The exceptions were items derived from COPSOQ´s cognitive and emotional demands scales, which included the original response scale: 1 = Never/Almost never; 2 = Seldom; 3 = Sometimes; 4 = Often; and 5 = Always. Note that for items concerning demands and unnecessary tasks, a low value is positive, and for the others, a high value is positive. The questions were formulated to capture the present (e.g., “Do you feel that …”) in line with suggestions for capturing experience data, and to minimize the risk of recollection bias [47].

### Psychometric testing

#### Setting

The DoCCA scale was tested in a Swedish primary care setting in which a pilot test of an eHealth service was conducted that entailed shifting tasks and activities from primary care providers to patients, such as for blood pressure measurements. The eHealth service consisted of monitoring devices and a smartphone application. Measurements, as well as trends and alerts, were automatically communicated to the individuals and their primary care staff. Asynchronous communication through chat was also supported.

#### Recruitment

Participants were recruited through the primary care organization. The eHealth service targeted adult patients (≥ 18 years of age) diagnosed with hypertension, chronic heart failure, or mental health conditions, including reaction to severe stress and adjustment disorders, insomnia, anxiety disorders, and depressive disorders. Individuals with one or more of these chronic conditions were eligible. Further inclusion criteria for participating in the pilot test were having a smartphone and email account and being able to communicate in Swedish, since this was the language used in the smartphone application.

Primary care staff identified patients that met the diagnostic inclusion criteria and called them by phone to inform them of the project. Interested and eligible participants were invited to a group enrollment session at the primary care clinic in September and October 2018. During the session, they were informed about the research project by a member of the research team and were invited to participate. Informed consent was obtained. The project followed the guidelines of the Helsinki Declaration and was approved by the Regional Ethical Review Board of Stockholm (reg nr. 2018/625-31/5 and 2018/1717-32).

#### Participants and data collection

Data were collected at two time points using a web-based questionnaire after enrollment in the eHealth project and seven months later. Thus, data were collected both when participants had recently been introduced to the eHealth service, hence assessing a co-care system during more traditional chronic care, and after they had personally used the eHealth service, allowing for validation of co-care in the same population with and without significant eHealth components. The questionnaire contained several measures (list available on demand). Here, we report those used in the psychometric testing of the DoCCA scale.

The questionnaire was distributed to 308 recipients. Reminders were sent out one and two weeks after the initial mailing. The response rate was 174 (56 %). The second questionnaire was distributed to the same 308 recipients after seven months. A total of 134 responded (after two reminders), yielding a response rate of 44 %. One-hundred and thirteen respondents (37 %) provided complete answers to both questionnaires. Of these, 81 (72 %) reported using the eHealth service to manage their hypertension, 21 (19 %) for mental conditions, 20 (18 %) for other (unspecified) chronic conditions, and nine (8 %) for heart failure. Four respondents (4 %) did not know what they used the eHealth service for. Twenty respondents (18 %) reported that they suffered from comorbid chronic conditions.

#### Statistical analysis strategy

The analysis included four parts. First, the psychometric properties of the DoCCA scale were investigated by evaluating construct validity with Confirmatory Factor Analysis (CFA). The CFA tested whether the items loaded on the intended dimension. Each set of items was allowed to load only on its corresponding latent variable. No correlation errors either within or across sets of items were allowed. However, in line with the theoretical assumptions of the measurement instrument, non-zero correlations between the factors were allowed. The proposed models assumed three factors: activities, needs support, and goal orientation, with the first additionally divided into sub-factors of demands, unnecessary tasks, and role clarity. This second-order (SO) structure was compared to three alternative solutions: (1) a one-factor (1 F) model with all items loading onto one factor, (2) a three-factor (3 F) model where demands, unnecessary tasks, and role clarity formed one factor (i.e., no sub-factors within activities) and needs support and goal orientation formed two separate first-order factors, and (3) a five-factor (5 F) solution with demands, unnecessary tasks, role clarity, needs support, and goal orientation as independent factors.

In line with the multifaceted approach to assessment of model fit, we considered the following fit indices: Comparative Fit Index (CFI) [48], Tucker and Lewis Index (TLI) [49], Root Mean Square Error of Approximation (RMSEA) [50], along with 90 % confidence interval limits, and (Standardized) Root Mean Square Residual ([S]RMR) [48]. We used the following values as thresholds recommended in the literature: TLI and CFI > .90 [51], RMSEA < .08 [50], and (S)RMR < .08. We followed the steps described for T1 data. Time 2 data served as a cross-validation, where we tested the goodness-of-fit indices of the model chosen in step 1. CFA analyses were performed in Mplus [52].

Second, the scale’s validity was examined. We computed Pearson’s moment correlations between the identified factors. To test concurrent validity, we computed cross-sectional correlations between the subscales of our instrument and a previously-validated measure of patients’ experiences managing a chronic condition, the six-item Self-Efficacy in Self-Care (SESSC) scale [53]. The SESSC was developed to measure self-efficacy in the context of minor illness. It includes questions such as: “*How certain are you that you can:”*, e.g., *“affect your symptoms?”*, *“regulate your activities so as to be active without aggravating your symptoms?”.* Responses were made on a four-point scale (1 = very uncertain and 4 = very certain). Self-efficacy and co-care as a distributed system of activities are both constructs, that intend to reflect people’s experiences of managing a chronic condition, but in different ways. While DoCCA reflects the individual’s experience of the co-care system, SESSC reflect the individual’s confidence in his or her own ability to manage the condition. Thus, we do not expect perfect correlation between these measures, but, overall, that individuals with high self-efficacy scores would also have positive experiences of the distribution of co-care activities, and vice versa.

Third, as a test of predictive validity, we tested correlations between the dimensions of the DoCCA scale measured at T1 with satisfaction with healthcare measured at T2. A one-item satisfaction question was used, based on the Swedish national patient survey: *“What is your overall rating of the care you have received at the [name of primary care center] during the past 6 months?”.* Responses were made on a five-point scale (1 = bad, 2 = reasonable, 3 = good, 4 = very good, 5 = excellent). The expectation was that a positive experience in DoCCA (i.e., low demands, low unnecessary tasks, high role clarity, high needs support, high goal orientation) at T1 will predict better satisfaction with healthcare services at T2. Kendall’s tau-b (*τ*_b_) correlation coefficient was computed since satisfaction was measured on an ordinal scale.

Fourth, reliability analyses of the subscales were conducted by assessing the internal consistency of the subscales by calculating Cronbach’s alpha coefficients. Test-retest reliability was analyzed by computing Pearson’s moment correlations for each subscale based on the sample of 113 respondents for whom both T1 and T2 data were available.

In all analyses, missing data were deleted list-wise.

## Results

### Confirmatory factor analyses

First, we tested for the multivariate normal distribution of the variables. The two-sided multivariate skew and kurtosis tests of fit were significant (at *p* < .001), which indicates that the normality assumption cannot be accepted. Consequently, we used the Maximum Likelihood Robust (MLR) estimator instead of Maximum Likelihood (ML) estimator to test the fit of the models. This estimator requires comparing the models using the Satorra-Bentler scaled chi-square test [54].

Next, the theoretically supported SO-model was tested against the alternative models. The results of the CFA regarding the goodness-of-fit indices are presented in Table 1.

**Table 1:** Results of the CFAs at T1

	Model			
	1F	3F	5F	SO
CFI	.65	.63	.94	.94
TLI	.61	.57	.92	.93
RMSEA [90 % CI]	.16 [.15–.17]	.17 [.16–.18]	.07 [.06–.09]	.07 [.06–.08]
(S)RMR	.14	.27	.08	.08
χ^2^	869	924	291	295
df	170	167	160	164
Scaling Correction Factor for MLR	1.24	1.22	1.20	1.20

*Note.1F* one-factor model (all items load into 1 factor); *3F *three-factor model (activities, needs support, goal orientation); *5F* five-factor model (demands, unnecessary tasks, role clarity, needs support, and goal orientation); *SO* second-order model (activities, needs support, and goal orientation, with activities having three subfactors: demands, unnecessary tasks, role clarity); *CFI* Comparative Fit Index; *TLI* Tucker and Lewis Index; *RMSEA* Root Mean Square Error of Approximation; *(S)RMR* (Standardized) Root Mean Square Residual

As Table 1 demonstrates, all goodness-of-fit indices exceeded the acceptable cut-off values for both Models 1F and 3F. Thus, neither 1F nor 3F adequately fit the data. Both the five-factor solution (5F) and the SO-model solution fitted the data significantly better than 1F and 3F (all *p*s < .001) and with acceptable goodness-of-fit indices. For the SO-model, all three first-order factors loaded significantly on the higher-level factor of the activities (*p* < .05). The SO-model did not differ significantly from 5F. With the models being inseparable statistically, the decision was instead made based on theory, with the SO-model being chosen as the best representation of the data. Table 2 presents factor loadings for this final solution at T1. All items loaded significantly on the first-order factors and exceeded the suggested minimum of .35 [55].

**Table 2:** Factor loadings of the second-order solution in the DoCCA scale

Factor	Item	Stand. Est.	SE	*Stand. Est/SE*	*p*
Demands	When you take care of your health, do you feel that:				
	... you need to make difficult decisions?	.82	.03	26.62	< .001
	... you need to remember a lot?	.88	.03	34.11	< .001
	... you need to keep track of many things at once?	.92	.02	45.45	< .001
	... you end up in emotionally demanding situations?	.85	.03	34.28	< .001
Unnecessary tasks	Do you feel that your self-care includes tasks that:				
	... actually do not need to be done?	.84	.05	17.51	< .001
	... do not really make sense?	.94	.05	20.37	< .001
	... could be done with less effort if healthcare were organized differently?	.55	.08	7.25	< .001
Role clarity	Do you feel that:				
	… responsibility is reasonably distributed between you and healthcare?	.82	.04	22.76	< .001
	… it is clear which areas are your responsibility?	.79	.04	18.71	< .001
	… it is clear which areas are healthcare’s responsibility?	.87	.03	25.12	< .001
	… you know what you can expect from healthcare?	.76	.08	9.78	< .001
	… you know what is expected of you from healthcare?	.74	.05	13.68	< .001
Needs support	Do you feel that:				
	… you are involved in decisions about your care and treatment as much as you wish?	.77	.04	19.29	< .001
	… you get the help and support you need to take care of your health?	.92	.02	48.22	< .001
	… you receive all the information you need to take care of your health?	.92	.02	45.26	< .001
	… the information you receive is clear?	.87	.03	30.84	< .001
Goal orientation	Do you feel that:				
	... healthcare knows what is important to you?	.81	.04	19.41	< .001
	... you and healthcare strive in the same direction?	.81	.04	19.19	< .001
	... you, together with healthcare, have agreed on goals for your health?	.88	.02	41.68	< .001
	... healthcare supports you in achieving your goals?	.89	.03	35.81	< .001
*Second order estimates*					
Activities	**Demands**	.26	.11	2.48	.013
	**Unnecessary tasks**	.32	.11	3.05	.002
	**Role clarity**	.94	.07	13.50	< .001

To cross-validate the chosen second-order solution, we performed a CFA on T2 data. Again, this solution reached acceptable values: CFI = .93, TLI = .92, RMSEA = .08 [.06–.09], (S)RMR = .07, and χ2 (164) = 281.

### Validity test

Table 3 demonstrates intercorrelations between subscales at T1 (below the diagonal) and at T2 (above the diagonal). Overall, the directions of the correlations are in line with theory and expectations and support the convergent validity of the scale. There were large positive correlations between role clarity, needs support, and goal orientation. Both high demands and unnecessary tasks were linked with lower role clarity, poorer needs support, and poor goal orientation. There were no substantial correlations between demands and unnecessary tasks. The results for T2 closely replicated those of T1.

**Table 3:** Subscale’s correlations, descriptive statistics, reliability, and inter–item correlations of the DoCCA scale (T1 and T2 samples)

	1. Demands	2. Unnecessary tasks	3. Role clarity	4. Needs support	5. Goal orientation
1. Demands	.73^***^	.04	− .23^*^	− .23^*^	−.26^**^
2. Unnecessary tasks	.11	.49^***^	− .27^**^	− .27^**^	− .18^*^
3. Role clarity	− .26^**^	− .37^***^	.67^***^	.84^***^	.77^***^
4. Needs support	− .18^*^	− .41^***^	.80^***^	.68^***^	.86^***^
5. Goal orientation	−.24^**^	− .37^***^	.76^***^	.83^***^	.65^***^
*Descriptives*					
*M* T1/T2	2.51/2.31	2.21/2.25	3.38/3.42	3.42/3.50	3.37/3.43
*SD* T1/T2	1.03/0.90	0.84/0.85	0.82/0.80	0.88/0.83	0.87/0.85
Cronbach’s Alpha T1/T2	.92/.90	.79/.86	.90/.90	.93/.92	.91/.92
Inter-item correlations (lowest–highest) T1/T2	.79–.87/.68–.87	.49–.77/ .64–.79	.70–.84/.68–.86	.74–.89/ .78–.88	.71–.82/ .78–.87

Note. Correlations below the diagonal show T1 relationships between variables. Correlations above the diagonal show T2 relationships between variables. Correlations on the diagonal show test-retest (T1-T2) correlations for the subscales

*N*_*T1*_ = 158, *N*_*T2*_ = 119, *N*_*T1−T2*_ = 113. * *p* < .05, ** *p* < .01, *** *p* < .001

To examine the DoCCA scale’s validity, we also tested correlations between the subscales and the SESSC. Both T1 and T2 correlations indicated a negative relation between SESSC and demands (r = –.46/–.43, respectively), i.e., the higher a patient’s self-efficacy, the fewer demands experienced in the management of the chronic disease. There was no significant relationship between SESSC and unnecessary tasks (r = –.11/.04, respectively). We detected low-to-medium positive relationships between SESSC and role clarity (r = .36/.29, respectively), needs support (r = .29/.29, respectively), and goal orientation (r = .29/.29, respectively).

As a test of predictive validity, we correlated T1 DoCCA dimensions’ scores with satisfaction with healthcare measured at T2. As expected, demands (*τ*_*b*_ = − .20, *p =* .010) and unnecessary tasks (*τ*_*b*_ = − .19, *p =* .017) were negatively linked with satisfaction with healthcare seven months later. Also, in line with our expectations, role clarity (*τ*_*b*_ = .32, *p* < .001), needs support (*τ*_*b*_ = .38, *p* < .001), and goal orientation (*τ*_*b*_ = .40, *p* < .001) were positively linked with subsequent satisfaction with healthcare.

### Reliability test

Cronbach’s alpha values for all dimensions of the DoCCA scale at T1 and T2 ranged between .79 and .93 (see Table 3), which suggests a good to excellent reliability of the subscales. We also examined the test-rest reliability of T1 and T2 responses by testing correlations between the two measurement points among individuals who completed both surveys (*N* = 113). As predicted, we observed positive and strong correlations (see Table 3), supporting the relative stability of the constructs.

## Discussion

This study proposed and tested an operationalization of the concept of co-care as a distributed system of activities in chronic care management. The theory of distributed cognition, which is recognized in human–computer interaction research but has received limited attention in health services research, was used to frame co-care as a sociotechnical system where chronic care activities are distributed between actors and artifacts and over time and space. Three co-care dimensions were defined: activities, goal orientation, and needs support. By focusing on the work of having a chronic condition and using work psychology as a lens, we suggest that the experience of the tasks distributed between individuals, healthcare, eHealth, and others may be understood in terms of perceived demands, unnecessary tasks, and role clarity. The psychometric evaluation supported the theoretical model and suggested that the proposed constructs can be reliably measured using the DoCCA scale.

### Theoretical and empirical considerations

The conceptualized second-order model was tested against three alternative solutions. The second-order model clearly fitted the data better than a one-factor model and a three-factor model; however, the single-order five-factor model also fitted the data well. This implies that the sub-factors are distinguishable but that they have a latent factor in common (i.e., activities). Based on its resonance with the theoretically derived model, the second-order model with demands, unnecessary tasks, and role clarity all loading significantly on the higher-order factor, activities, is the best representation of co-care as a distributed system of activities. The final model and scale had consistently stable psychometric properties, including all items loading significantly on the expected first-order factor, exceeding the suggested minimum of .35 [55]. Thus, the findings demonstrate that demands, unnecessary tasks, role clarity, needs support, and goal orientation are empirically distinct, albeit correlated, factors.

To assess how individuals perceive the activities, needs support, and goal orientation, instruments that previously have been used in a work context were applied to assess how employees perceive their working conditions. For *activities*, we found scales measuring demands (emotional and cognitive), unnecessary tasks, and role clarity applicable to the experience of co-care [44, 45]. For *needs support*, some promising items were found in the work psychology and patient experience literature but no whole scales [44, 46]. The items were amended to reflect the system as a whole rather than individual parts of the system. Work psychology generally refers to support from managers and colleagues and the patient experience literature to support from healthcare professionals. We aimed to make the needs support scale neutral to the source of the support to determine whether the individual receives sufficient support independently of who provides it. For example, by asking if the individual receives all the information needed, it is acknowledged that the information revolution and social media advancements mean that individuals access information from multiple sources [56]. The needs support subscale was further amended to reflect the degree to which the individual’s *needs* for support are met, which is different from the degree to which support is *offered.* Although the needs support subscale was not based on any specific theory, it seems reflective of the universal psychological needs as defined in the self-determination theory, particularly autonomy and competence [57]. The applicability of self-determination theory for needs support in co-care merits further exploration in future research.

The intention of the *goal orientation* subscale was to reflect how well system actors understand, support, and/or are aligned with what matters to the individual for whom the system exists. This made scales from work psychology less applicable because in a work context, it is the organization’s goals that are the focal point of the system. However, surprisingly, many patient experience measures *also* fell short of meeting the requirement of having the individual with the chronic condition as a focal point. They tended to ask about *patients’* involvement in healthcare, thus viewing the patient-healthcare relationship from the perspective of healthcare, not the opposite [18]. Similarly, when the importance of power sharing and involvement in decision making is emphasized, it is generally the healthcare provider that is asked to share power with the patient [14]. Co-care takes the opposite perspective. This may reflect one of the fundamental differences between co-care as a distributed system of activities and other patient experience concepts.

### When and where can the scale be used?

The DoCCA scale can be used to assess individuals’ experiences of their chronic care system regardless of which components (actors and artifacts) the system contains. A satisfactory co-care situation occurs when there is a combination of tasks being distributed in a way that makes the load acceptable (low demands, few unnecessary tasks, clear roles), there is sufficient needs support, and there is an alignment and orientation of actions towards what matters to the individual. Thus, the purpose of the scale is not to evaluate specific parts of the system but rather how the system as a whole is experienced. It does not describe what the system looks like or how the different parts of the system contribute to the experience of co-care. The DoCCA scale can instead be used to study this empirically, e.g., which distributions of activities and which degrees of involvement are linked to positive co-care experiences. However, some subscales may be less applicable in contexts that are highly fragmented. Rating the system as a whole may be challenging if the individual has conflicting experiences of the different parts of the system, e.g., high goal orientation in relation to one part of healthcare but low to another.

The fact that the scale does not reflect a certain way of organizing chronic care means that the scale can be used to understand how different initiatives contribute to individuals’ overall experiences of their chronic care, e.g., how shared decision making, eHealth services, person-centeredness, and co-production and co-creation initiatives affect the overall experience of co-care. For example, how much healthcare involvement is necessary for individuals to experience co-care? Which factors explain variations in this relationship? Under which conditions does shared decision making lead to increased co-care?

The scale can also be used for evaluating and/or comparing initiatives aimed to optimize chronic care management as long as the initiatives imply a shift in roles or responsibilities between actors and artifacts in the system. For example, the DoCCA scale can be used to assess how individuals experience task shifting from healthcare professionals to patients, which may follow from the introduction of eHealth services, or to investigate whether satisfaction with co-care is higher with shared decision making compared to unilateral decision making. In these cases, the scale can capture the degree to which changes in the chronic care service lead to an improvement in individuals’ experiences of co-care. Because the scale is neutral to the specific content of the innovation, the DoCCA scale can be used in combination with other scales that focus on evaluating specific innovations or specific aspects, such as a certain episode (e.g., a shared decision making situation) or relationship.

The DoCCA scale does not assume that a certain way to design the co-care system is better than another. One individual may perceive co-care as satisfactory when he or she does most him- or herself with limited involvement from healthcare, and another may experience the same situation as unsatisfactory, instead requiring more healthcare involvement to report satisfactory co-care. Thus, the DoCCA scale can be used to explain variations in outcomes of interventions aiming to improve chronic care management. For example, an eHealth service may only be related to positive outcomes if the demands on the individual are perceived as reasonable and fair. Therefore, in combination with other measures, the DoCCA scale may support the identification of variations in preferences and may contribute to the development of healthcare services and non-healthcare services that best suit the preferences among individuals with chronic conditions.

### Methodological considerations

We refined the co-care concept by applying the theory of distributed cognition and operationalized it based on how perceptions of “work” are understood and assessed in a work context. This resulted in the theoretical model (Fig. 1), which was tested with CFA, an approach appropriate for testing whether data are consistent with a pre-understanding of a construct [58]. Rather than a particular data set “dictating” underlying dimensions, the CFA approach requires that the researchers theorize an underlying structure and assess whether the observed data “fits” this a priori specified model. We replicated the solution from the first analysis using data from T2. However, it should be noted that there is an overlap between the respondents in the two samples (113 of 174 and 134, respectively, responded at both time points). Thus, the model should be cross-validated using an independent sample.

Convergent, concurrent and predictive validity of the scale were, overall, satisfactory with significant correlations in the expected directions, with the exception of the correlation between unnecessary tasks and demands, and unnecessary tasks and self-efficacy. The latter indicates that perceived demands and unnecessary tasks capture complementary aspects of the distribution of work within the co-care system. The correlation between DoCCA subscales and self-efficacy was overall low to medium, which may indicate that although they both intend to reflect patients’ experience of managing a chronic condition, they assess different aspects, supporting the suggestion that the DoCCA scale may complement rather than substitute other patient experience measures. Further research on the relationship between DoCCA and other patient experience constructs is warranted.

The test-retest reliability was assessed by testing correlations across time points with panel data and was satisfactory, however, there was a change in the components of the co-care system between the two measurement time points. This is not optimal for testing test-retest reliability, as this means that two tests are not done under the same conditions. The different conditions for the retest also limit the possibility to go beyond reliability to also test agreement, since this cannot be assumed when the conditions are not the same [59]. Nevertheless, to provide more nuance to the reliability test about the stability of the scale, we created Bland-Altman plots for the five subscales (see Additional file 1). These plots showed that over 90 % of the participants across the five subscales are within the limits of agreement (+/-2SD), and the graphs indicate a stable behavior of the measurement. There were no particular trends except for demands, where participants reporting low values at T1 seemed slightly more likely to report an increase at T2. Thus, further evaluations of the scale in new samples are warranted. Nevertheless, by testing the scale with individuals with limited experiences of an eHealth service and then again after it was introduced into the co-care system, the applicability of the DoCCA scale to different configurations of co-care systems was supported.

This study was conducted in primary care in Sweden, limited to individuals speaking Swedish. Also, the scale was originally developed in Swedish and translated to English for this publication, calling for further investigation of the English version, in new populations and contexts. Participants were individuals diagnosed with hypertension, chronic heart failure, or mental health conditions, eligible for testing the new eHealth service. Therefore, a broad range of chronic conditions typically managed in primary care were represented, so the applicability across different conditions was considered in the development of the scale, focusing on the commonalities between individuals with a chronic condition that they manage with the support of primary care, other actors, and artifacts. Yet, the applicability to other conditions and settings merits further investigation.

The aspiration of the DoCCA scale was to make it neutral vis-à-vis which actor performs which activity. This was successful for demands, needs support, and three of four items in unnecessary tasks; however, goal orientation and role clarity (in three of five items) all refer to healthcare. The direct reference to healthcare reflects the central role that healthcare plays in the setting for this study: because it was conducted in collaboration with a primary care center, they were a specified part of the co-care system. In addition, attempts to formulate the items neutrally, such as with reference to “the system,” were unsuccessful, possibly because respondents are not accustomed to the term in this context. This means that whereas demands, unnecessary tasks, and needs support can be used to assess systems that do not include healthcare, the goal orientation and role clarity items may need to be revised to reflect actors other than healthcare.

Lastly, the DoCCA scale focuses exclusively on the experience of the individual with a chronic condition. Future development may include corresponding scales for healthcare professionals or other actors in a co-care system.

## Conclusions

As self-care is becoming the focal point of chronic care management, healthcare professionals and eHealth services have become supporting nodes in a sociotechnical system. This study contributes to the current understanding of chronic care management and patient experiences by applying a system perspective founded in the theory of distributed cognition and inspired by how the perception of work is conceptualized in the domain of work psychology. With this approach, chronic care management is conceptualized as work that has to be done, involving the individual supported by, for example, healthcare professionals and eHealth services, with fluent boundaries between healthcare and self-care.

The co-care concept, as measured by the DoCCA scale, offers a way to assess individuals’ experiences in managing their chronic conditions in the light of the sociotechnical system that is available to them. It may be thought of as a thermometer, taking the temperature as to what degree the system (1) is oriented to what matters to the individual, (2) provides sufficient support for achieving it, and (3) distributes the activities in a way that is manageable for the individual. As such, it complements concepts and instruments that target specific parts of the system, such as a certain eHealth services, the relationship with healthcare staff, or shared decision making.

## Supplementary Information

**Additional file 1**

## Data Availability

The datasets used and/or analyzed during the current study are available from the corresponding author on reasonable request.

## Abbreviations

BITS: Bern Illegitimate Tasks Scale
CFA: Confirmatory Factor Analysis
CFI: Comparative Fit Index
COPSOQ: Copenhagen Psychosocial Questionnaire
DoCCA: Distribution of Co-Care Activities
ML: Maximum Likelihood
MLR: Maximum Likelihood Robust
RMSEA: Root Mean Square Error of Approximation
SALAR: Swedish Association of Local Authorities and Regions
SESSC: Self-Efficacy Scale in Self-Care
SO: Second-Order
TLI: Tucker and Lewis Index
T1: Timepoint 1
T2: Timepoint 2
1F: One-factor
3F: Three-factor
5F: Five-factor

## References

[1] Glasgow RE, Davis CL, Funnell MM, Beck A (2003). Implementing Practical Interventions to Support Chronic Illness Self-Management. Jt Comm J Qual Saf.

[2] Nelson EC, Meyer G, Bohmer R (2014). Self-care: The new principal care. J Ambul Care Manage.

[3] Wagner EH (1998). Chronic disease management: what will it take to improve care for chronic illness?. Eff Clin Pract.

[4] Eysenbach G (2001). What is e-health?. J Med Internet Res.

[5] Beck J, Greenwood DA, Blanton L, Bollinger ST, Butcher MK, Condon JE, et al. 2017 National Standards for Diabetes Self-Management Education and Support. Diabetes Educ. 2017 Jul 28;43(5):449–64. 10.1177/0145721717722968 28753378

[6] Sheppard JP, Schwartz CL, Tucker KL, McManus RJ (2016). Modern Management and Diagnosis of Hypertension in the United Kingdom: Home Care and Self-care. Ann Glob Heal.

[7] Wannheden C, Revenäs Å. How People with Parkinson’s Disease and Healthcare Professionals Wish to Partner in Care Using eHealth: Co-design Study. J Med Internet Res 2020;22(9):e19195 Available from: https://www.jmir.org/2020/9/e19195. 10.2196/19195 10.2196/19195 PMC7536604 32955448

[8] Von Thiele Schwarz U. Co-care: Producing better health outcome through interactions between patients, Care providers and information and communication technology. Heal Serv Manag Res [Internet]. 2016;29(1–2):10–5. Available from: 10.1177/0951484816637746

[9] Ropohl G. Philosophy Of Socio-Technical Systems. Techné Res Philos Technol. 1999 Mar 22;4:186–94.

[10] Wolf JA, Niederhauser V, Marshburn D, Lavela SL (2014). Defining patient experience. Patient Exp J.

[11] LaVela S, Gallan A. Evaluation and Measurement of Patient Experience. Patient Exp J [Internet]. 2014;1(1):28–36. Available from: http://pxjournal.org/journal/vol1/iss1/5

[12] Lindwall M, Weman-Josefsson K, Sebire SJ, Standage M. Viewing exercise goal content through a person-oriented lens: A self- determination perspective. Psychol Sport Exerc [Internet]. 2016;27:85–92. Available from: 10.1016/j.psychsport.2016.06.011

[13] Scholl I, Kriston L, Dirmaier J, Buchholz A, Härter M (2012). Development and psychometric properties of the Shared Decision Making Questionnaire - physician version (SDM-Q-Doc). Patient Educ Couns.

[14] Lindberg J, Kreuter M, Person LO, Taft C (2013). Patient Participation in Rehabilitation Questionnaire (PPRQ) - Development and psychometric evaluation. Spinal Cord.

[15] Moses H, Matheson DHM, Dorsey ER, George BP, Sadoff D, Yoshimura S (2013). The anatomy of health care in the United States. JAMA - J Am Med Assoc.

[16] Silvera G, Haun C, Wolf J. Patient Experience: The field and future. Patient Exp J. 2017 Apr 25;4:7–22.

[17] Drewes HW, de Jong-van Til JT, Struijs JN, Baan CA, Tekle FB, Meijboom BR, et al. Measuring chronic care management experience of patients with diabetes: PACIC and PACIC + validation. Int J Integr Care. 2012;12:e194. 10.5334/ijic.862 10.5334/ijic.862 PMC3601510 23593054

[18] Wolf JA (2019). Reframing the conversation on patient experience: Three considerations. Patient Exp J.

[19] Kremer JAM, Van Der Eijk M, Aarts JWM, Bloem BR. The individual formerly known as patient, TIFKAP. Minerva Med. 2011;102(6):505. 22193382

[20] Hutchins E (1995). Cognition in the wild.

[21] Lippa KD, Feufel MA, Robinson FE, Shalin VL. Navigating the decision space: Shared medical decision making as distributed cognition. Qual Health Res [Internet]. 2017;27(7):1035–48. Available from: 10.1177/1049732316665347 10.1177/1049732316665347 27557927

[22] Petrakaki D, Hilberg E, Waring J. Between empowerment and self-discipline: Governing patients’ conduct through technological self-care. Soc Sci Med [Internet]. 2018;213:146–53. Available from: http://www.sciencedirect.com/science/article/pii/S0277953618304106 10.1016/j.socscimed.2018.07.043 PMC6137078 30081356

[23] Kuutti K. The concept of activity as a basic unit of analysis for CSCW research. In: Bannon L., Robinson M. SK, editor. Proceedings of the Second European Conference on Computer-Supported Cooperative Work ECSCW ’91. Dordrecht: Springer; 1991.

[24] Karlgren K, Paavola S, Ligorio MB. Introduction: what are knowledge work practices in education? How can we study and promote them? Res Pap Educ [Internet]. 2020 Jan 2;35(1):1–7. Available from: 10.1080/02671522.2019.1677761

[25] Spector PE, Jex SM (1998). Development of four self-report measures of job stressors and strain: Interpersonal Conflict at Work Scale, Organizational Constraints Scale, Quantitative Workload Inventory, and Physical Symptoms Inventory. J Occup Health Psychol.

[26] Demerouti E, Bakker AB, Nachreiner F, Schaufeli WB. The job demands-resources model of burnout. Vol. 86, Journal of Applied Psychology. US: American Psychological Association; 2001. p. 499–512. 11419809

[27] Semmer NK, Tschan F, Meier LL, Facchin S, Jacobshagen N (2010). Illegitimate tasks and counterproductive work behavior. Appl Psychol.

[28] Siegrist J (1996). Adverse health effects of high-effort/low-reward conditions. Vol. 1, Journal of Occupational Health Psychology.

[29] Snow R, Humphrey C, Sandall J. What happens when patients know more than their doctors? Experiences of health interactions after diabetes patient education: a qualitative patient-led study. 2013;1–8. 10.1136/bmjopen-2013-003583 PMC3831109 24231459

[30] Holman H, Lorig K. Patient Self-Management: A Key to Effectiveness and Efficiency in Care of Chronic Disease. 2004;119(June):239–43. 10.1016/j.phr.2004.04.002 PMC1497631 15158102

[31] Kamal AH, Kirkland KB, Meier DE, Morgan TS, Nelson EC, Pantilat SZ. A Person-Centered, Registry-Based Learning Health System for Palliative Care: A Path to Coproducing Better Outcomes, Experience, Value, and Science. J Palliat Med [Internet]. 2017 Nov 1;21(S2):S-61-S-67. Available from: 10.1089/jpm.2017.0354 10.1089/jpm.2017.0354 PMC5756463 29091509

[32] Berwick DM (2009). What “patient-centered” should mean: Confessions of an extremist. Health Aff.

[33] Langford CPH, Bowsher J, Maloney JP, Lillis PP (1997). Social support: A conceptual analysis. J Adv Nurs.

[34] Kriston L, Scholl I, Hölzel L, Simon D, Loh A, Härter M. The 9-item Shared Decision Making Questionnaire (SDM-Q-9). Development and psychometric properties in a primary care sample. Patient Educ Couns [Internet]. 2010;80(1):94–9. Available from: 10.1016/j.pec.2009.09.034 10.1016/j.pec.2009.09.034 19879711

[35] Fan J, McCoy RG, Ziegenfuss JY, Smith SA, Borah BJ, Deming JR, et al. Evaluating the Structure of the Patient Assessment of Chronic Illness Care (PACIC) Survey from the Patient’s Perspective. Ann Behav Med [Internet]. 2015 Feb 1;49(1):104–11. Available from: 10.1007/s12160-014-9638-3 10.1007/s12160-014-9638-3 25236671

[36] Griffiths F, Cave J, Boardman F, Ren J, Pawlikowska T, Ball R, et al. Social networks – The future for health care delivery. Soc Sci Med [Internet]. 2012;75(12):2233–41. Available from: http://www.sciencedirect.com/science/article/pii/S0277953612006302 10.1016/j.socscimed.2012.08.023 22985490

[37] Swan M (2009). Emerging patient-driven health care models: An examination of health social networks, consumer personalized medicine and quantified self-tracking. Int J Environ Res Public Health.

[38] Elwyn G, James P, Grande SW, Thompson R, Walsh T, Ozanne EM. Patient Education and Counseling Developing CollaboRATE: A fast and frugal patient-reported measure of shared decision making in clinical encounters. Patient Educ Couns [Internet]. 2013;93(1):102–7. Available from: 10.1016/j.pec.2013.05.009 10.1016/j.pec.2013.05.009 23768763

[39] Hibbard JH, Stockard J, Mahoney ER, Tusler M (2004). Development of the patient activation measure (PAM): Conceptualizing and measuring activation in patients and consumers. Health Serv Res.

[40] Légaré F, Moher D, Elwyn G, Leblanc A, Gravel K. Instruments to assess the perception of physicians in the decision-making process of specific clinical encounters: A systematic review. BMC Med Inform Decis Mak. 2007 Feb 1;7:30. 10.1186/1472-6947-7-30 PMC2151936 17937801

[41] Lewis JR. The System Usability Scale: Past, Present, and Future. Int J Human–Computer Interact [Internet]. 2018 Jul 3;34(7):577–90. Available from: 10.1080/10447318.2018.1455307

[42] Orwelius L, Nilsson M, Nilsson E, Wenemark M, Walfridsson U, Lundström M (2018). The swedish rand-36 health survey-reliability and responsiveness assessed in patient populations using svensson’s method for paired ordinal data. J Patient-Reported Outcomes.

[43] Schön U-K, Svedberg P, Rosenberg D. Evaluating the INSPIRE measure of staff support for personal recovery in a Swedish psychiatric context. Nord J Psychiatry [Internet]. 2015 May 19;69(4):275–81. Available from: 10.3109/08039488.2014.972453 10.3109/08039488.2014.972453 25377024

[44] Kristensen T, Hannerz H, Hogh A, Borg V. The Copenhagen Psychosocial Questionnaire - A tool for the assessment and improvement of the psychosocial work environment. Scand J Work Environ Health. 2006 Jan 1;31:438–49. 10.5271/sjweh.948 16425585

[45] Semmer NK, Jacobshagen N, Meier LL, Elfering A, Beehr TA, Kälin W, et al. Illegitimate tasks as a source of work stress. Work Stress [Internet]. 2015 Jan 2;29(1):32–56. Available from: 10.1080/02678373.2014.1003996 10.1080/02678373.2014.1003996 PMC4396521 25892839

[46] Swedish Association of Local Authorities and Regions (SALAR). Flippen i primärvården. Ett nationellt innovationsprojekt med och för sveriges primärvård - slutrapport [Internet]. 2018. Available from: https://docplayer.se/108184770-Flippen-i-primarvarden-ett-nationellt-innovationsprojekt-med-och-for-sveriges-primarvard-slutrapport-flippen-i-primarvarden-1.html

[47] Schwarz N, Stone AA, Shiffman S, Nebeling L (2007). Retrospective and concurrent self-reports: the rationale for real-time data capture. The science of real-time data capture: Self-reports in health research.

[48] Bentler PM (1990). Comparative fit indexes in structural models. Psychol Bull.

[49] Bagozzi R, Yi Y. On the Evaluation of Structure Equation Models. J Acad Mark Sci. 1988 Jan 26;16:74–94.

[50] Browne MW, Cudeck R. Alternative Ways of Assessing Model Fit. Sociol Methods Res [Internet]. 1992 Nov 1;21(2):230–58. Available from: 10.1177/0049124192021002005

[51] Hu LT, Bentler PM, Hoyle RH (1995). Evaluating model fit. Structural equation modeling Concepts, issues, and applications.

[52] Muthén LK, Muthén BO, Mplus (2017). Statistical Analysis with Latent Variables: User’s Guide (Version 8).

[53] Gustafsson S, Sävenstedt S, Vikman I (2013). Psychometric evaluation of a Swedish self-efficacy scale and recovery locus of control scale in the context of minor illness. Scand J Caring Sci.

[54] Bryant FB, Satorra A. Principles and Practice of Scaled Difference Chi-Square Testing. Struct Equ Model A Multidiscip J [Internet]. 2012 Jul 20;19(3):372–98. Available from: 10.1080/10705511.2012.687671

[55] Costello AB, Osborne J. Best Practices in Exploratory Factor Analysis: Four Recommendations for Getting the Most From Your Analysis. Pract Assessment, Res Eval. 2005 Jan 1;10:1–9.

[56] Knaup P, Ammenwerth E, Dujat C, Grant A, Hasman A, Hein A, et al. Assessing the Prognoses on Health Care in the Information Society 2013 - Thirteen Years After. J Med Syst [Internet]. 2014;38(7):73. Available from: 10.1007/s10916-014-0073-6 10.1007/s10916-014-0073-6 24952606

[57] Ryan RM, Deci EL. Overview of self-determination theory: An organismic-dialectical perspective. In: Ryan ELDRM, editor. Handbook of self-determination research. University of Rochester Press.; 2002. p. 3–33.

[58] Kline RB (2010). Principles and practice of structural equation modeling.

[59] Berchtold A. Test–retest: Agreement or reliability? Methodol Innov [Internet]. 2016 Jan 1;9:2059799116672875. Available from: 10.1177/2059799116672875

