# Supplementary material for: The work of having a chronic condition: development and psychometric evaluation of the distribution of co-care activities (DoCCA) scale
Source: BMC Health Serv Res. 2021 May 20;21:480. doi: 10.1186/s12913-021-06455-8 (PMC8138998; doi:10.1186/s12913-021-06455-8)

# Bland-Altman analyses for DoCCA T1-T2

To compare two measurements of DoCCA dimensions across the two timepoints (T1 and T2), we generated Bland-Altman (mean-difference or limits of agreement) plots. These plots show the difference between two observations on the vertical axis against the average of the two observations on the horizontal axis. The thick line represents mean difference, and the dashed lines represent 95% limits of agreement (average difference ± 1.96 standard deviation of the difference). All analyses are for *N* = 113.

Overall, these plots demonstrate that the differences do not vary in any systematic way over the range of measurement across the dimensions. Demands could be considered an exception, where for low values participants seemed more likely to report an increase, whereas for the high values – a decrease.

## Unnecessary tasks


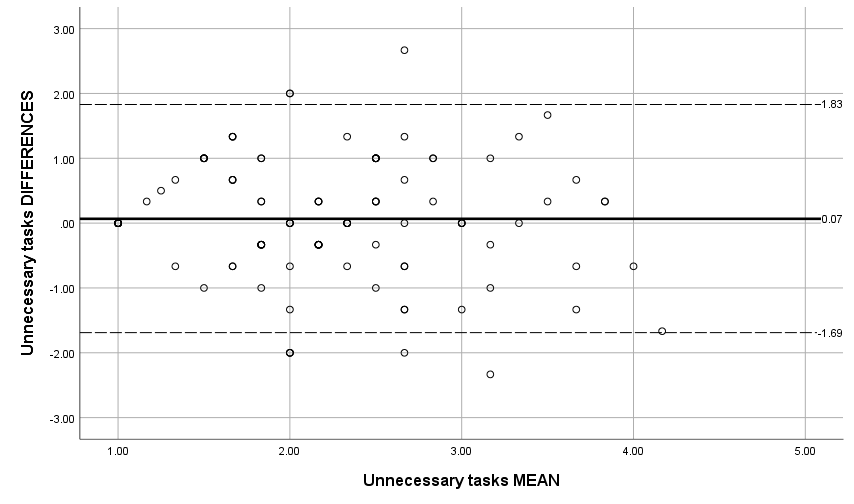


## Demands


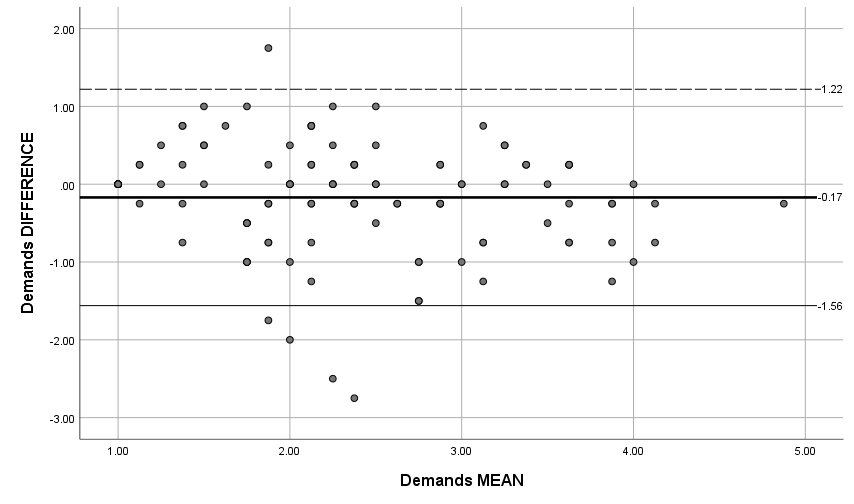


## Role clarity


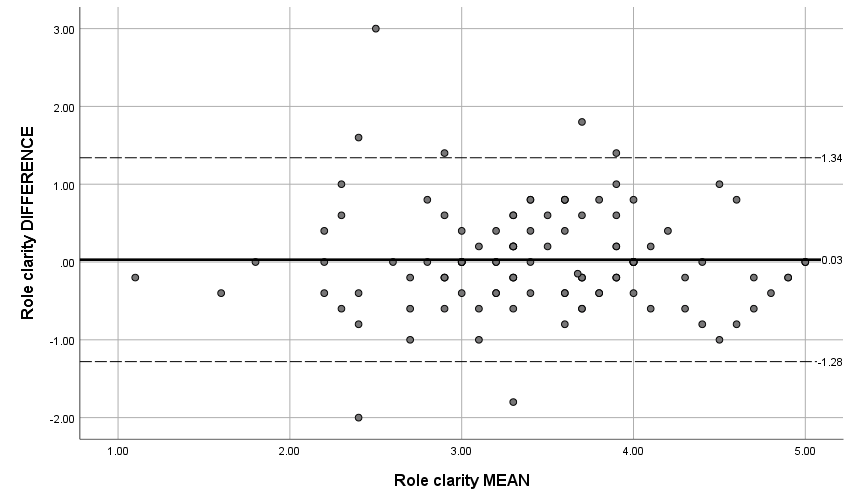


## Needs support


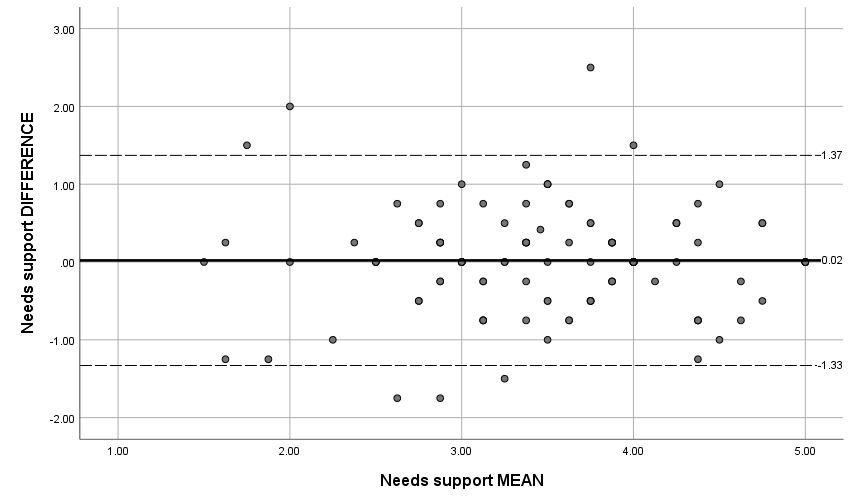


## Goal orientation


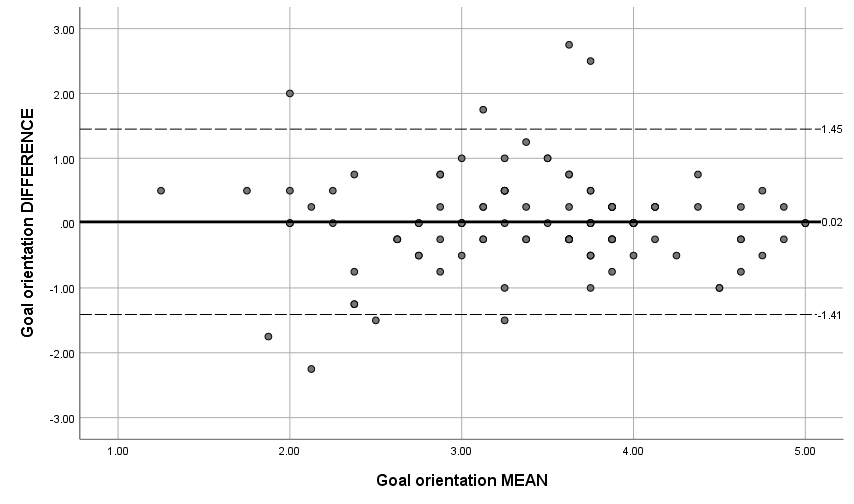

Supplement: Supplementary file 1 — Additional file 1 [file 12913_2021_6455_MOESM1_ESM.docx]
